# Alcohol’s Effects on Sleep in Alcoholics

**Published:** 2001

**Authors:** Kirk J. Brower

**Affiliations:** Kirk J. Brower, M.D., is an associate professor of psychiatry at the University of Michigan Alcohol Research Center and executive director of the Chelsea Arbor Treatment Center, Ann Arbor, Michigan

**Keywords:** sleep disorder, AOD (alcohol or other drug) dependence, physiological AODE (effects of AOD use, abuse, and dependence), REM (rapid eye movement) sleep, AOD withdrawal syndrome, AOD abstinence, self medication, AODD (AOD use disorder) relapse, melatonin, treatment and maintenance, literature review

## Abstract

Sleep problems, which can have significant clinical and economic consequences, are more common among alcoholics than among nonalcoholics. During both drinking periods and withdrawal, alcoholics commonly experience problems falling asleep and decreased total sleep time. Other measures of sleep are also disturbed. Even alcoholics who have been abstinent for short periods of time (i.e., several weeks) or extended periods of time (i.e., several years) may experience persistent sleep abnormalities. Researchers also found that alcoholics are more likely to suffer from certain sleep disorders, such as sleep apnea. Conversely, sleep problems may predispose some people to developing alcohol problems. Furthermore, sleep problems may increase the risk of relapse among abstinent alcoholics.

Sleep problems^1^ are more common among alcoholics than among non-alcoholics (Aldrich 1998; Ehlers 2000; National Institute on Alcohol Abuse and Alcoholism [NIAAA] 1998). For example, in the general population, insomnia in the previous 6 months affected 18 percent of alcoholic people, but only 10 percent of nonalcoholic people (Brower et al. 2000). Among patients admitted for alcoholism treatment, rates of insomnia are even higher, ranging from 36 percent to 72 percent, depending on sample characteristics, the instrument used to measure sleep, the amount of time elapsed since the last drink, and the presence of other disorders (i.e., comorbidity) (see table 1).

Alcohol-related sleep problems, however, encompass more than symptoms of insomnia. For the purposes of this article, the term “sleep problems” is used interchangeably with the terms “sleep disturbances,” “sleep abnormalities,” “impaired sleep,” and “disrupted sleep.” All of these terms refer both to subjective complaints about sleep, such as insomnia, and to objectively measured abnormalities in sleep, which can be determined using a procedure called polysomnography. (This technique is described in more detail in the following section.)

The consequences of sleep problems in alcoholics are economically and clinically significant. Overall, Stoller (1994) estimated that approximately 10 percent of all alcohol-related costs could be attributed to insomnia. Given that alcohol problems cost the United States an estimated $184.6 billion in 1998 (NIAAA 2000), alcohol-related insomnia may have cost the country around $18.5 billion that year. Furthermore, sleep apnea—a sleep disorder characterized by recurrent episodes of breathing cessation that occurs more frequently in alcoholics than in nonalcoholics—has been associated with increased mortality from heart disease and stroke (Aldrich et al. 1999). Other potential consequences of sleep disturbances, which can be exacerbated by alcoholism, include impaired daytime performance (Roth and Ancoli-Israel 1999), memory dysfunction (Roehrs and Roth 1995), and increased risk for depression (Gillin 1998; Weissman et al. 1997). In the general population, insomnia has been associated with premature mortality after controlling for physical health and other risk factors (e.g., Pollak et al. 1990), although other studies did not find that effect (e.g., Althuis et al. 1998).

This article first describes briefly the various sleep stages that researchers have identified and how they are measured. It then reviews alcohol’s effects on the sleep of alcoholics, including effects observed during active drinking, acute alcohol withdrawal, and sustained sobriety. The discussion continues with the potential relationship between sleep problems and the development of alcoholism as well as the possible role of sleep disturbances in predicting relapse to alcoholism. The article concludes by exploring treatment implications of these findings.

## Sleep Stages and their Measurement

The various sleep stages and their characteristics are described in more detail in the accompanying article by Roehrs and Roth, pp. 101–109 of this issue. Briefly, sleep is generally classified into two sleep states, rapid eye movement (REM) sleep and nonrapid eye movement (NREM) sleep, which alternate in several cycles throughout the night. As the name implies, REM sleep is characterized by frequent movements of the eyes and is typically associated with dreaming. During NREM sleep, the eyes typically do not move in a rapid fashion; this sleep state is further divided into four stages determined by polysomnography, which is described in the following paragraph. Stages 1 and 2 are sometimes referred to as light sleep, because it is relatively easy to awaken people during these stages. Stages 3 and 4 collectively are called deep sleep, or slow-wave sleep (SWS), because it is difficult to awaken people during these stages.

The gold standard for objectively measuring sleep is polysomnography. This strategy measures numerous variables, including breathing characteristics, eye movements, leg movements, percentage of time spent in each sleep stage, sleep continuity (i.e., sleep latency, total sleep time, and sleep efficiency), and REM sleep latency. The term “sleep latency” refers to the time between going to bed and sleep onset. Similarly, the term “REM sleep latency” refers to the time between sleep onset and the onset of the first episode of REM sleep. The term “sleep efficiency” refers to the proportion of time in bed that is spent sleeping. Other variables used to characterize sleep are the percentage of total sleep time spent in REM sleep (i.e., REM%) and in SWS (i.e., SWS%), respectively.

## Sleep in Alcoholics During Experimental Drinking and Acute Withdrawal

**Figure 1:**
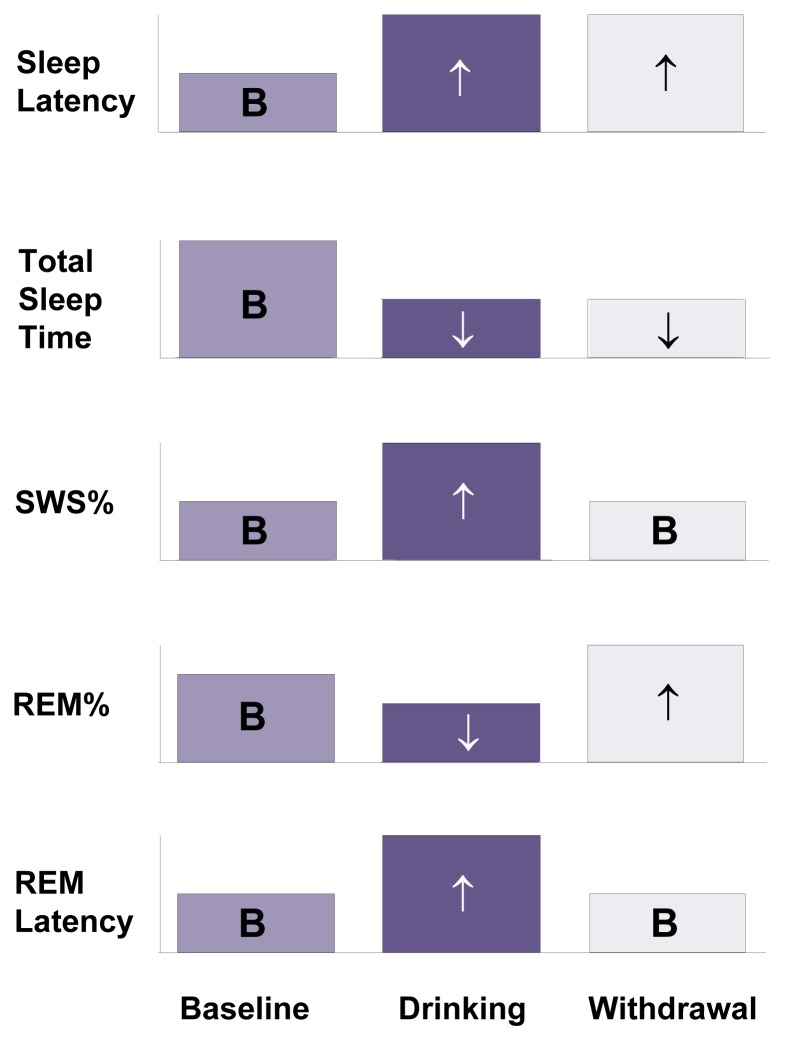
A summary of nocturnal sleep changes in alcoholic patients as determined across various polysomnographic studies of acute alcohol administration and withdrawal. The studies measured sleep characteristics at baseline, after drinking, and during acute alcohol withdrawal. Note that the size of the bars indicates only the *direction*, but not the *magnitude*, of the changes. Both after drinking and during withdrawal, sleep latency increases and total sleep time decreases, compared with the response at baseline. Both the percentage of deep sleep, or slow-wave sleep (SWS), and the rapid eye movement (REM) sleep latency increase during drinking and return to baseline levels during withdrawal. Although SWS% returns to baseline values during withdrawal, researchers should note that baseline values of SWS% in alcoholics are still lower than values from control subjects. REM% decreases with drinking and then returns to or even exceeds baseline levels during withdrawal. NOTES: Sleep latency is the time between going to bed and actually falling asleep. SWS% is the proportion of deep sleep, or SWS, during total sleep time. REM% is the proportion of REM sleep during total sleep time. REM latency is the time between sleep onset and the onset of the first episode of REM sleep.

Several research groups in the 1970s and early 1980s used polysomnography to study the sleep of male alcoholics undergoing inpatient alcoholism treatment, both following alcohol administration and during alcohol withdrawal (Allen et al. 1980; Gross et al. 1973; Gross and Hastey 1975; Wagman and Allen 1975; Zarcone 1978).^2^ In most of these studies, alcohol administration to alcoholic patients resulted in difficulty falling asleep (i.e., prolonged sleep latency), decreased total sleep time, increased SWS%, decreased REM%, and increased REM sleep latency (see figure 1). Only one study (Allen et al. 1980) found an increased total sleep time after alcohol administration to alcoholic patients.^3^ In addition, Gross and Hastey (1975) noted that the baseline SWS% of alcoholic patients ranged widely from 0.7 to 44 percent and that alcohol-induced increases in SWS% depended on the baseline values of SWS%. Thus, when baseline levels of SWS% were less than 20 percent, heavy drinking produced either no change or a decrease in SWS%. Conversely, when baseline levels of SWS% were between 20 and 40 percent, heavy drinking resulted in an increase in SWS%.

When the sleep of alcoholics was studied during withdrawal, sleep latency remained increased and total sleep time remained decreased compared with baseline levels (see figure 1). In contrast, SWS% and REM sleep latency decreased during withdrawal relative to drinking nights and returned to baseline levels. Finally, REM% increased during withdrawal and even exceeded baseline levels, a phenomenon called REM rebound. One small study of three alcoholic men who received alcohol (7.6 ounces of pure alcohol) for 4 to 7 days assessed sleep characteristics over several days of withdrawal (Allen et al. 1971). In that study, REM% decreased during the first 2 to 3 days of withdrawal and then rebounded by days 5 and 6. However, this pattern of the effects of withdrawal on REM% has not been reported since.

Research results suggest that although some variability exists across studies, the following general conclusions can be drawn regarding sleep measures in alcoholics:

- Measures of sleep continuity (i.e., sleep latency and total sleep time) are disrupted on both drinking and withdrawal nights in alcoholic patients. The finding of increased sleep latency contrasts with decreases in sleep latency found in healthy (i.e., nonalcoholic) men after drinking alcohol and suggests that alcoholic patients develop tolerance to the sleep-inducing effects of alcohol but remain sensitive to its stimulating effects.
- SWS% increases during drinking and returns to baseline levels during withdrawal.
- REM sleep generally is suppressed during drinking and either rebounds (with respect to REM%) or returns to baseline levels (with respect to REM latency) during withdrawal.

Two other studies assessed sleep during acute withdrawal without using polysomnography. Mello and Mendelson (1970) made behavioral observations of sleep among 40 male inpatients during a 5-day baseline period and a subsequent drinking period averaging 14 days during which most subjects drank from 12.5 to 16.0 ounces of pure alcohol each day. This drinking period was followed by a 3- to 6-day withdrawal period. The authors defined insomnia as sleeping less than the minimum number of sleep hours observed during baseline. Using this criterion, 25 percent of the patients (i.e., 10 patients) developed insomnia during the first 48 hours of withdrawal, and a total 58 percent of the patients (i.e., 23 patients) had at least 1 night of insomnia during the first 6 days of withdrawal. Closer inspection of the data indicated that in 5 of those 23 patients, the insomnia was possibly related to discontinuing medications for detoxification. Thus, in 18 of the 40 patients (i.e., 45 percent), the insomnia was associated with alcohol withdrawal. The authors concluded that insomnia was not an invariable finding during withdrawal from alcohol (see table 1). In addition, Caetano and colleagues (1998) investigated the prevalence of insomnia as an acute withdrawal symptom among 748 men admitted to detoxification and residential treatment centers. The analysis, which used a structured interview to assess insomnia, found that 67 percent of the men reported insomnia. The findings of these two studies complement polysomnographic studies of acute alcohol withdrawal that found evidence of insomnia as indicated by increases in sleep latency and decreases in total sleep time.

The most severe manifestation of alcohol withdrawal is delirium tremens (DTs), which is characterized by tremors, agitation, and hallucinations. Several studies found that DTs are associated with fragmented sleep—that is, frequent awakenings or arousals that alternate with episodes of light sleep (i.e., stage 1) or REM sleep (Johnson et al. 1970; Greenberg and Pearlman 1967; Gross et al. 1966). Immediately after an episode of DTs, light sleep predominates and REM sleep decreases, as indicated by significantly decreased SWS% and REM% and increased percentages of stages 1 and 2 sleep, compared with control subjects (Kotorii et al. 1982). The frequent juxtaposition of waking, light sleep, and REM sleep during DTs, as well as the decrease in REM sleep that follows an episode of DTs, supports early theories that the hallucinations of DTs represent an intrusion of REM sleep processes into the waking state (for a review, see Zarcone 1978).

## Sleep in Alcoholics During Postwithdrawal Abstinence

The acute withdrawal phase after cessation of alcohol consumption lasts approximately 1 to 2 weeks. Some withdrawal-like symptoms, such as insomnia, craving, and mood instability, however, persist even beyond that period, a phenomenon variously called “subacute withdrawal,” “protracted abstinence,” or “protracted withdrawal” (Gross and Hastey 1976; Satel et al. 1993). This section discusses persisting sleep disturbances during both recent (i.e., lasting 2 to 8 weeks) and sustained (lasting more than 3 months) abstinence.

### Recent Abstinence

To assess sleep problems associated with recent abstinence, Alling and colleagues (1982) interviewed 56 alcoholic patients who had been abstinent for 2 to 60 days. The investigators concluded that insomnia was a common subacute withdrawal symptom that persisted for approximately 5 weeks. Although the relevance of this study is somewhat limited because it lacked a control group and objective measures of sleep, its results are consistent with studies that did use nonalcoholic control subjects and polysomnography. In such studies, alcoholic patients who had been abstinent for 2 to 8 weeks exhibited worse sleep than did nonalcoholics (Aldrich et al. 1999; Gillin et al. 1990*b*; Le Bon et al. 1997; Williams and Rundell 1981). In those studies, total sleep time, sleep efficiency, and SWS% were generally decreased significantly, whereas stage 1 sleep usually was increased (Aldrich 1998; Le Bon et al. 1997). In addition, sleep latency was significantly increased in most studies of abstinent alcoholics (Benca et al. 1992).

Some controlled studies also reported increased REM sleep and shortened REM sleep latency in recently sober alcoholics (Gillin et al. 1990*a*; Moeller et al. 1993; Williams and Rundell 1981), although other studies found no effects on REM sleep (Aldrich et al. 1999; Gillin et al. 1990*b*; Le Bon et al. 1997). These inconsistent findings regarding REM sleep may reflect differences in alcoholic subtypes in the different studies. Thus, persistent REM sleep abnormalities were most evident in depressed alcoholics (Gillin et al. 1990*a*; Moeller et al. 1993) and alcoholics who subsequently relapsed (Gillin et al. 1994; Brower et al. 1998). However, further studies are required to clarify this issue.

In addition to psychiatric comorbidity, such as depression, researchers have identified other variables that can influence sleep measures in recently abstinent alcoholic patients. These factors include age, gender, ethnicity, medical problems, other drug use, quantity of drinking and duration of heavy drinking, time since last drink, and severity of alcohol withdrawal and dependence (Brower and Hall 2001; Gillin et al. 1990*b*; Irwin et al. 2000; Wetter and Young 1994).

### Sustained Abstinence

Polysomnographic analyses found that some sleep abnormalities can persist for 1 to 3 years after cessation of alcohol consumption (see table 2). For example, two study groups reported more frequent than normal shifting from one sleep stage to another, suggesting sleep “fragmentation,” after 12 to 24 months of abstinence (Adamson and Burdick 1973; Williams and Rundell 1981). Two other indicators of sleep fragmentation (i.e., brief arousals and REM sleep disruptions) also persisted throughout 21 months of abstinence (Williams and Rundell 1981). Although sleep latency appeared to normalize by 5 to 9 months of abstinence, total sleep time took 1 to 2 years to return to normal levels (Adamson and Burdick 1973; Drummond et al. 1998).

The persistence of REM sleep abnormalities during prolonged abstinence varies across studies, as follows:

- Drummond and colleagues (1998) found that REM% remained increased, and REM sleep latency remained decreased after 27 months of abstinence.
- Schiavi and colleagues (1995) reported no significant differences in REM sleep time and REM sleep latency when comparing 20 alcoholic men with 2 to 36 months of sobriety with control subjects.
- Williams and Rundell (1981) noted that REM% normalized by 9 months, whereas REM sleep latency was persistently shortened at 9 months of abstinence.
- Imatoh and colleagues (1986) found that the distribution of REM sleep across the night changed significantly with increasing duration of sobriety. In healthy people, the majority of REM sleep occurs during the last third of the night. In alcoholics abstinent for 10 days or 1 month, however, the majority of REM sleep occurred during the first two-thirds of the night, suggesting an abnormal phase advance in REM sleep; only at 3 months of abstinence did the distribution of REM sleep normalize. The authors speculated that chronic drinking and early stages of withdrawal produced an alteration in the circadian pattern of REM sleep that reversed by 3 months of sobriety.

Several studies assessing abnormalities in SWS% during prolonged sobriety indicated that SWS% remained suppressed for 3 to 14 months (Drummond et al. 1998; Imatoh et al. 1986; Ishibashi et al. 1987; Williams and Rundell 1981). Two longitudinal studies that followed alcoholics over several years found that SWS% normalized by 21 to 27 months (Williams and Rundell 1981; Drummond et al. 1998). These results are consistent with the findings of two cross-sectional studies, which reported normal SWS% between 1 and 4 years (Adamson and Burdick 1973; Wagman and Allen 1975).

In conclusion, sleep fragmentation manifested by increases in sleep-stage changes, brief arousals, and REM sleep disruptions can persist for 1 to 3 years after establishing sobriety. Furthermore, most sleep disturbances that occur during recent abstinence (i.e., decreased total sleep time and SWS%, and increased sleep latency and stage 1 sleep) appear to normalize with sustained abstinence. REM sleep latency may remain abnormal from 9 to 27 months. Although some studies documented a return to normal REM% at 3 to 9 months, REM% may remain elevated for 27 months.

When interpreting those results, however, one must consider several limitations of sustained-abstinence sleep studies, as follows:

- Many polysomnographic studies of sustained abstinence are based on relatively small sample sizes (see table 2).
- The vast majority of studies include only men; accordingly, little is known about the course of sleep abnormalities in abstinent women.
- Few studies specified the methodology for determining and verifying abstinence. Thus, some persistent sleep problems could reflect unrecognized drinking.
- With the exception of one study (Drummond et al. 1998), none of the studies used validated measures to exclude patients with sleep disorders (e.g., sleep apnea or periodic limb movement [PLM] disorder) or comorbid mental disorders. Thus, non-alcohol-related sleep abnormalities may have confounded some of the findings.
- Several studies included no control group, and of those studies that did include control subjects, none followed the control group longitudinally, even though sleep patterns change with age (Brower and Hall 2001).
- Mounting evidence indicates that alcoholic patients with good prognoses sleep better than do patients at a high risk for relapse. Therefore, studies of long-term abstinence may select for good sleepers and underestimate sleep problems. This is supported by the findings of Drummond and colleagues (1998), who noted that disrupted sleep at 5 months predicted relapse at 14 months; this drastically reduced the sample size over the course of the study.

Two common sleep disorders are sleep apnea and PLM disorder. Sleep apnea is diagnosed in part by recording (through polysomnography) the number of apnea episodes per hour of sleep to generate an apnea index. An apnea index of more than 5, although not diagnostic by itself, is more common among patients with sleep apnea than among other people. PLM disorder is characterized by repetitive jerking of the legs and sometimes arms during sleep. These movements can cause multiple arousals during the night, which can result in insomnia or daytime sleepiness.

Three studies assessed the prevalence of sleep apnea in recently sober alcoholics (Le Bon et al. 1997; Mamdani et al. 1989; Tan et al. 1985). The studies included a total of 116 participants, of whom 29.3 percent (i.e., 34 patients) had an apnea index greater than 5. Unfortunately, only one study (Tan et al. 1985) calculated the proportion of control subjects with an apnea index greater than 5. However, the number of those control subjects (i.e., 12 persons, out of whom none had an apnea index greater than 5) was too small to provide an adequate comparison across the three studies.

Aldrich and colleagues (1999) used a different measure of sleep-disordered breathing called an apnea-hypopnea index (AHI) to investigate sleep disorders in alcoholics and nonalcoholics. The study found that significantly more alcoholic than nonalcoholic subjects exhibited mild sleep-disordered breathing, as indicated by an AHI of 5 or more. The proportions of alcoholic and control subjects with clinically significant breathing problems (i.e., an AHI of 10 or more), however, did not differ.

The levels of oxygen in the blood are another indicator of whether breathing is interrupted during sleep. Vitiello and colleagues (1990) found that low levels of oxygen in the blood (i.e., hypoxemia) occurred more commonly in alcoholic men who had been abstinent for a mean of 115 days than in control subjects. In the alcoholic group, neither the number of abstinent days nor smoking predicted hypoxemia. Overall, these studies indicate that alcoholic patients even during abstinence may be more likely than control subjects to have sleep-disordered breathing, but specific results may vary depending on the particular measure and cut-off values used.

Three studies to date compared recently abstinent alcoholic patients and nonalcoholic control subjects with respect to PLMs. In one study, PLMs were significantly increased in 20 alcoholic men who had been abstinent for 2 to 36 months (Schiavi et al. 1995). In another study, PLMs were significantly higher in 139 alcoholic subjects who had been abstinent for a mean of 1 month than in 87 control subjects (Brower and Hall 2001). Conversely, Le Bon and colleagues (1997) found an absence of PLMs both in alcoholic subjects who had been abstinent for 3 to 6 weeks and in nonalcoholic subjects.

## Development of Alcohol and Sleep Problems

The fact that some sleep problems observed among alcoholics may persist despite sustained abstinence suggests three possible explanations:

- Some sleep disturbances could precede the development of alcoholism and thus persist into abstinence.
- Chronic alcoholism may cause either slowly reversible or irreversible damage to brain systems that regulate sleep.
- Chronic alcoholism may be associated with persisting medical and psychiatric disorders that disrupt sleep during abstinent periods.

**Figure 2:**
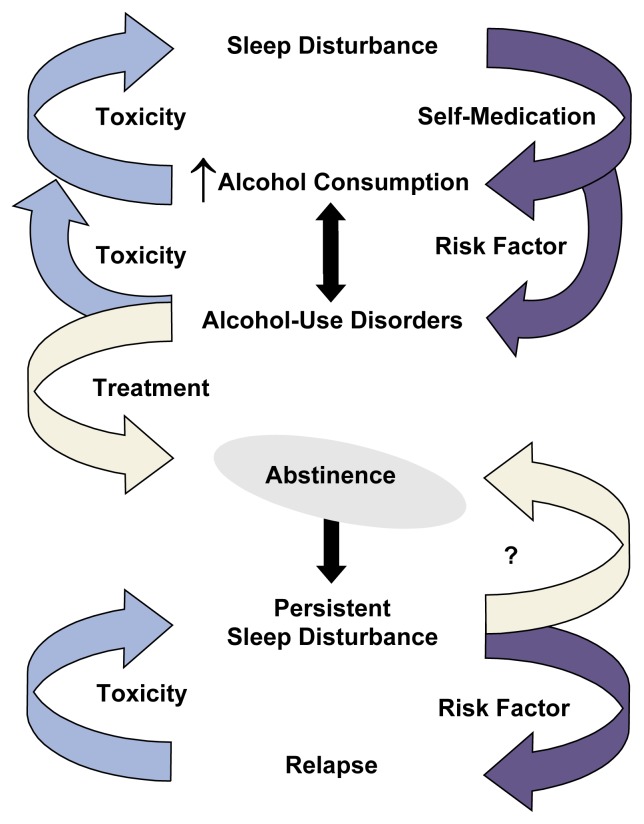
A model of the reciprocal relationships between heavy alcohol consumption and sleep disturbances. Sleep disturbance may lead to increased alcohol consumption for self-medication. At the same time, alcohol consumption, through its effects on brain chemicals (i.e., neurotoxicity), may lead to sleep disturbance. Sleep disturbance is also a risk factor for developing alcohol-use disorders (i.e., alcohol abuse and alcohol dependence). Treatment of these disorders can lead to abstinence, but sleep disturbances may persist even during recent and sustained abstinence. Sleep disturbances at the time of treatment are risk factors for relapse to drinking. In turn, relapse contributes to alcohol neurotoxicity and persistent sleep disturbances. The question mark represents the untested hypothesis that treatment of sleep disturbances as an adjunct to alcoholism treatment can facilitate abstinence and decrease the risk of relapse. Purple arrows indicate processes that favor unhealthy patterns of drinking, blue arrows indicate processes that favor sleep disturbance, and yellow arrows represent treatment processes that may favor abstinence.

Although the potential relationships between alcohol consumption and insomnia are complex, several recent reviews have described a reciprocal relationship (Blumenthal and Fine 1996; NIAAA 1998; Vitiello 1997). Specifically, insomnia may lead to initial and recurrent problem drinking, and heavy alcohol consumption may disrupt sleep and contribute to insomnia (see figure 2). These two relationships are discussed in the following sections.

### Can Sleep Problems Predispose People to Alcoholism?

Three epidemiological reports have addressed the issue of whether a history of insomnia can predict the development of alcohol abuse or dependence. A 1989 study by Ford and Kamerow (see Gillin 1998) used data collected during the Epidemiological Catchment Area survey, a national household survey. The investigators reported that in the general population, the incidence of alcohol abuse was 2.4 times higher in adults who experienced persistent insomnia during the previous year than in adults who had not.

The interpretation of these findings is somewhat limited, however, because the analysis did not exclude people who had other psychiatric disorders prior to the survey that might have contributed to the alcohol abuse. To address this issue, Weissman and colleagues (1997) further analyzed the data after excluding respondents with psychiatric disorders prior to the survey. These investigators calculated that adults with insomnia had a significantly increased likelihood (i.e., an odds ratio of 2.3) of developing alcohol abuse compared with adults without insomnia.

In the third study, investigators also demonstrated a trend (i.e., an odds ratio of 1.72) for new-onset alcohol abuse or dependence following a history of insomnia; however, the numbers were not statistically significant (see Gillin 1998). Nevertheless, the results of the three studies suggest that insomnia precedes the development of alcohol problems in at least some adults.

People commonly use alcohol to self-medicate for sleep problems. Some studies have estimated that 6 to 19 percent of the general population and 15 to 28 percent of people with insomnia have used alcohol to promote sleep (Brower et al. 2001; Johnson et al. 1998; National Sleep Foundation 2000). To assess the use of alcohol as self-medication for sleep problems more thoroughly, Roehrs and colleagues (1999) studied 20 nonalcoholic adults in the laboratory, 11 of whom had insomnia and 9 of whom were normal sleepers. For the first 4 nights of the study, the participants received either alcoholic (i.e., a total of 0.5 grams alcohol per kilogram bodyweight, given in three divided doses over 45 minutes) or three nonalcoholic beverages that could be distinguished by color-coded cups. For the next 3 nights, the participants were allowed to choose their bedtime beverage from the color-coded cups. The investigators found that insomniacs chose the alcoholic beverage on 67 percent of the nights, whereas the normal sleepers chose the alcoholic beverage on 22 percent of the nights. Moreover, alcohol reduced self-reported feelings of tension in people with insomnia compared with normal sleepers (Roehrs et al. 1999). These epidemiological and laboratory studies suggest the possibility that self-medication of insomnia with alcohol could contribute to the development of alcohol problems in some people.

Alcohol use to self-medicate for sleep problems is even more common among alcoholics: between 44 and 60 percent of alcoholic patients report using alcohol to help them sleep prior to entering treatment (Brower et al. 2001). However, alcohol is not a reliably effective sedative among alcoholic patients. For example, as discussed earlier, alcohol may prolong sleep latency and decrease total sleep time in alcoholics (see figure 1). An inpatient study of 56 alcoholic men assessed alcohol’s effect on subjective sleep quality during a 1-week baseline period during which the men received no alcohol and a subsequent 4-week period during which the men could choose to drink (Skoloda et al. 1979). The study found that those participants who chose to drink had worse sleep quality at baseline than did participants who did not drink. Furthermore, alcohol temporarily improved sleep quality in the drinking patients; however, this effect was confined primarily to the first week of drinking. These results indicate that alcoholic patients may rapidly develop tolerance to alcohol’s sedative effects, rendering it ineffective as a sleep aid.

Several animal studies addressed the possibility that sleep disturbances in early development could lead to heavy alcohol consumption later in life (e.g., Hilakivi et al. 1987). In these studies, newborn rats were treated with various antidepressants, resulting in reduced levels of REM sleep compared with untreated rats. As adults, the treated rats exhibited decreased levels of certain brain chemicals implicated in sleep (i.e., monoamine neurotransmitters) and increased alcohol consumption compared with the untreated rats.

### Can Sleep Problems Predict Relapse Among Alcoholics?

#### Review of Studies

Allen and Wagman (1975) were the first investigators to provide experimental evidence that objectively measured sleep abnormalities were possibly associated with relapse among abstinent alcoholics. In that study, four alcoholic men underwent polysomnography for at least 7 nights after undergoing inpatient detoxification. Each subsequent morning, the men’s predisposition to drink was tested via a behavioral reinforcement test. In that test, the men could press a button repeatedly to obtain an alcoholic drink within 3.5 hours; by pressing the button more frequently, they could obtain the drink earlier. For example, the men could obtain the drink after 3.5 hours by pressing the button 300 times or after 1 hour by pressing the button 3,300 times. The results indicated that low REM% was correlated with a high frequency of button pressing (i.e., a high predisposition to drink). In a related study of six alcoholic men, however, Allen and Wagman (1975) could not demonstrate that experimental deprivation of REM sleep increased the predisposition to drink.

Nevertheless, a potential association between the levels of REM sleep and alcohol intake is also supported by animal studies. For example, REM-sleep deprivation did increase alcohol intake in rats, an effect possibly mediated by the brain chemical (i.e., neurotransmitter) norepinephrine (Aalto and Kiianmaa 1986). (For more information on the neurobiology of alcoholism and sleep problems, see the sidebar, pp. 118–123.) Furthermore, alcohol-preferring rats have lower levels of REM% compared with non-alcohol-preferring rats (Aalto and Hilakivi 1986).

**The Neurobiology of Alcoholism and Sleep Problems**

As mentioned in the main article, brain chemicals (i.e., neurotransmitters) that mediate the transmission of nerve signals from one nerve cell (i.e., neuron) to the next likely play important roles in mediating alcohol’s effect on sleep in alcoholics. These neurotransmitters are released by the signal-emitting neuron, travel across a small gap (i.e., the synapse) between the neurons, and then interact with protein molecules (i.e., receptors) on the surface of the signal-receiving neurons. For some neurotransmitters, more than one type of receptor exists. Depending on the type of neurotransmitter (and, in some cases, the type of receptor), receptor activation can either result in or prevent the generation of a new nerve signal or the production of new proteins in the signal-receiving neuron. Neurotransmitters that allow the generation of a new nerve signal are called stimulatory neurotransmitters, whereas those that prevent the generation of a new nerve signal are called inhibitory neurotransmitters.

Limited research has been conducted on the neurobiological mechanisms underlying the effects of chronic, heavy alcohol consumption on sleep (for a review, see Ehlers 2000). Substantial knowledge exists, however, regarding the neurobiology of alcoholism (Koob and Roberts 1999; Valenzuela and Harris 1997) and the neurobiology of sleep (Aldrich 1999; Jones 2000). Therefore, the neurobiological mechanisms that both influence sleep and are affected by alcohol provide a starting point for exploring the reciprocal relationships between alcoholism and sleep (see table). The following sections review some of these relationships.

***Neurotransmitter Systems Involved in Sleep***

Early theories regarding the neurobiology of sleep focused on the role of a group of compounds called monoamine neurotransmitters, particularly serotonin and norepinephrine (Jouvet 1969). Because alcohol also was known to affect these compounds, investigators speculated that alcohol disrupted sleep by altering the actions of monoamine neurotransmitters (Johnson et al. 1970; Smith et al. 1971; Zarcone et al. 1978). Consistent with this hypothesis, an early study reported that alcohol-related sleep disturbances in men improved with 5-hydroxytryptophan, a medication that enhances serotonin functioning (see Zarcone 1978). Conversely, a later study failed to find that a similar agent, the serotonin precursor L-tryptophan, improved sleep any more than did an inactive substance (i.e., a placebo) in detoxified alcoholic patients (Asheychik et al. 1989). In the latter study, however, the subjects had not been selected based on sleep complaints, and they slept relatively well prior to receiving medication, which may have distorted the research results.

Nevertheless, the simple notion that reduced levels of serotonin function explain poor sleep in alcoholics is unlikely. Although the precise role of serotonin systems in sleep regulation is unknown (Aldrich 1999), the following evidence suggests that serotonin activation is not necessary for normal sleep (Jones 2000):

- Certain neurons that secrete serotonin and which are located in the area of the brain stem called the raphe nucleus are most active during the waking state, decrease their firing rate during slow-wave sleep (SWS), and become even more inactive during rapid eye movement (REM) sleep. (For a description of the different sleep stages, see the main article as well as the article in this issue by Roehrs and Roth, pp. 101–109.)
- Serotonin release generally is lower during sleep than during waking.
- Serotonin can have both excitatory and inhibitory effects, depending on the type of receptor it interacts with on the signal-receiving cell.
- Medications that interfere with the actions of a type of serotonin receptor called 5HT2c (i.e., which act as receptor antagonists), such as the agent ritanserin, increase SWS in humans (Sharpley et al. 1994).
- No single neurotransmitter system is sufficient for ensuring normal sleep. Rather, normal sleep depends on a complex interplay of numerous neurotransmitter systems and sleep factors (Aldrich 1999), many of which are also affected by alcohol.

In general, the waking state is mediated by a system of neurons called the ascending reticular activating system and by the excitatory activities of numerous neurotransmitters, including norepinephrine, serotonin, dopamine, acetylcholine, histamine, and glutamate. Nonrapid eye movement (NREM) sleep, especially SWS, results from both a decrease in these excitatory neural systems and an increase in inhibitory neural activity. For example, both sleep-generating (i.e., hypnogenic) neurons located in a brain region called the basal forebrain and brain stem neurons containing the neurotransmitter gamma-aminobutyric acid (GABA) act to inhibit the ascending reticular activating system (Jones 2000). During REM sleep, the monoamine neurotransmitter and histamine systems further decrease in activity, whereas the acetylcholine system, and possibly the glutamate system, becomes activated (Aldrich 1999; Prospero-Garcia et al. 1994).

***Alcohol’s Effects on Sleep-Related Neurotransmitter Systems***

Acute alcohol administration affects all of the neurotransmitter systems mentioned in the previous section (see table below). For example, alcohol can enhance GABA activity, which is inhibitory, and can inhibit glutamate activity, which is stimulatory. Thus, these two actions could account for some of alcohol’s sedative properties. Glutamate inhibition might also mediate some of alcohol’s REM-suppressing effects (Prospero-Garcia et al. 1994). In addition, REM suppression could result from decreased activity of the acetylcholine system, because alcohol inhibits acetylcholine release in the brain (Valenzuela and Harris 1997).

**Table:**
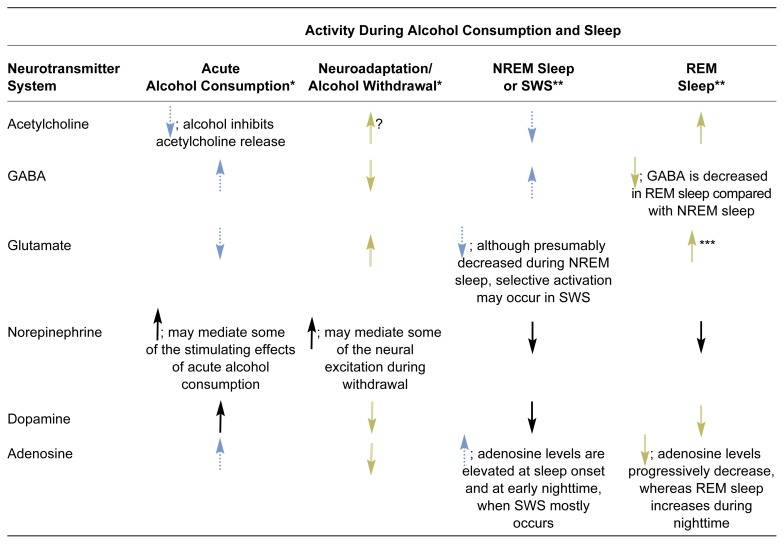
Putative Neuronal System Activity During Alcohol Consumption and Sleep

GABA = gamma-aminobutyric acid; NREM = nonrapid eye movement; REM = rapid eye movement; SWS = slow wave sleep.

NOTES: Blue-dotted arrow pairs in a given row indicate alcohol’s acute actions that promote NREM sleep. Yellow-open arrow pairs indicate neuroadaptation to alcohol of the neurotransmitter systems and withdrawal effects that favor REM rebound (i.e., greater-than-normal levels of REM sleep) during withdrawal. Neuroadaptation to alcohol of various neurotransmitter systems (with the exception of dopamine) also favors arousal and neural excitation.

* SOURCES: Becker 1999; Koob and Roberts 1999; Littleton 1998; Valenzuela and Harris 1997.

** SOURCES: Aldrich 1999; Jones 2000.

*** SOURCE: Prospero-Garcia et al. 1994.

Norepinephrine is another neurotransmitter that possibly mediates some of alcohol’s acute effects on sleep. For example, mice that have been genetically modified to lack norepinephrine are hypersensitive to alcohol’s sedative effects (Weinshenker et al. 2000), suggesting that intact norepinephrine systems oppose the sedative effects of alcohol (Munoz et al. 1986). Additional evidence regarding the role of norepinephrine derives from two other strains of selectively bred mice—long-sleep and short-sleep mice—which differ in their sedative response to alcohol. As the names imply, long-sleep mice have longer sleep times than short-sleep mice following acute exposure to alcohol. At the age when the animals’ differential sleep responses emerge, long-sleep mice have lower basal norepinephrine levels in their brain stems than do short-sleep mice (French et al. 1995), suggesting that norepinephrine activity provides some protection against alcohol-induced sedation in short-sleep mice. Furthermore, low alcohol doses, which can be stimulating in humans (Roehrs and Roth 1995), have been shown to raise nor-epinephrine levels in the cortex of rats (Rossetti et al. 1992). Conversely, higher alcohol doses, which can be sedating in humans, have been shown to lower norepinephrine release in rats. Thus, the dose-dependent effects of alcohol on sleep seem to parallel the dose-dependent effects of alcohol on norepinephrine release. Possibly, although still speculative, norepinephrine may mediate the increase in sleep latency and the decrease in total sleep time observed after acute alcohol administration to alcoholic patients (see figure 1 on p. 112, of the main article).

***Neuroadaptation, Alcohol Withdrawal, and Sleep***

Chronic alcohol consumption also results in long-term alterations and neuroadaptation in the neurotransmitter systems affected by alcohol, and these alterations persist into the early stages of abstinence (Becker 1999; Koob and Roberts 1999; Littleton 1998). Neuroadaptation means that in response to the chronic exposure to alcohol, the brain adjusts its baseline activities to compensate for alcohol’s effects on brain-cell functioning. For example, because alcohol tends to enhance GABA activity and inhibit glutamate activity, neuroadaptation to chronic alcohol consumption includes decreased baseline activity of inhibitory GABA systems and increased activity of excitatory glutamate systems. These alterations compensate for alcohol’s effects, allowing the brain to maintain its “normal” activity levels in the presence of alcohol. When alcohol is discontinued, however, these alterations persist, at least for a while, resulting in increased arousal that manifests as withdrawal symptoms, including sleep disruption. In general, neuroadaptation to chronic alcohol consumption and the resulting abnormal neurotransmitter activity during alcohol withdrawal favor central nervous system arousal and thus interfere with sleep-generating mechanisms.

Evidence that the GABA system is involved in the sleep disruptions of alcohol withdrawal has been found in studies using agents that mimic GABA’s actions on its receptor (i.e., GABA_A_ agonists). Treatment with such agents during withdrawal should compensate for the reduced baseline activity of GABA that occurs as a result of neuroadaptation. Consistent with this hypothesis, treatment with GABA_A_ agonists improved sleep during alcohol withdrawal in rats (Rouhani et al. 1998). Similarly, the GABA_A_ agonist diazepam increased polysomnographically measured total sleep time in alcoholic humans (Aubin et al. 1994).

Another excitatory neurotransmitter whose activity is altered by alcohol and which may contribute to withdrawal symptoms is noradrenaline. Noradrenaline activity is enhanced during alcohol withdrawal (Hawley et al. 1985), which likely contributes to increased arousal during the early withdrawal phase. This hypothesis is supported by findings that agents that reduce noradrenaline activity (e.g., benzodiazepines, clonidine, and propanolol) both have sedative effects and are useful in the treatment of alcohol withdrawal (Carskadon et al. 1989; Lejoyeux et al. 1998; Mayo-Smith 1997).

***The Role of Neuronal Cell Loss***

Early explanations for decreased SWS in alcoholic patients focused not on alcohol’s effects on individual neurotransmitter systems but on alcohol-induced diffuse damage to the brain’s cortex (Smith et al. 1971) and on alcohol-related loss of neurons (Adamson and Burdick 1973). Researchers developed this hypothesis after observing shrinkage (i.e., atrophy) of various brain regions in alcoholic patients. Consistent with this hypothesis, Ishibashi and colleagues (1987) demonstrated that improvement in SWS during a 6-month period among nine alcoholic men was associated with the reversal of atrophy in a certain brain region (i.e., the cerebrum) as measured by computed tomography (CT) scanning.

***Sleep Factors***

Several so-called sleep factors also have been implicated in the initiation and maintenance of sleep. These substances, which are naturally produced by the body, are released by certain cells into the bloodstream or the fluid surrounding the brain (i.e., cerebrospinal fluid) and can induce sleep. One sleep factor that may mediate some of alcohol’s effects on sleep is growth hormone-releasing hormone (GHRH) (Krueger et al. 1999; Lands 1999). Episodic administration of GHRH promotes SWS in both animals and humans. As the name implies, GHRH also stimulates the release of growth hormone, although the sleep-promoting action of GHRH does not depend on growth hormone.

Another sleep factor that might mediate alcohol-related sleep disturbances is adenosine, a neuromodulator that by itself does not generate new nerve signals but which modulates the ability of other neurotransmitters to generate new nerve signals. Adenosine has sleep-promoting inhibitory effects on the central nervous system, including the acetylcholine system, which it exerts at adenosine receptor sites (Jones 2000).

Caffeine stimulates wakefulness by blocking adenosine receptors. Brain levels of adenosine increase with prolonged waking and before sleeping, suggesting that it may have a role in sleep induction (see table in sidebar).

Acute alcohol administration enhances adenosine activity (Koob and Roberts 1999), which in turn inhibits the acetylcholine system. Because acetylcholine contributes to REM sleep, alcohol-induced increases in adenosine activity may play a role in decreasing REM sleep following alcoholic intoxication. Conversely, during alcohol withdrawal, adenosine activity is lower than normal, which favors arousal and excessive REM sleep (i.e., REM rebound). Finally, proteins produced by the immune system (i.e., cytokines) have known effects on sleep and are altered in alcoholic individuals (Ehlers 2000; Krueger et al. 1999).

***Summary***

Numerous neurotransmitter systems and other substances are involved in the regulation of sleep and various sleep stages. Both acute and chronic alcohol consumption alter the activity of many of these neurotransmitters—such as serotonin, norepinephrine, GABA, glutamate, and noradrenaline—as well as affect other sleep factors. These alterations may contribute to the sleep disturbances observed both in alcoholics and in people undergoing alcohol withdrawal. For the most part, however, the specific mechanisms underlying the relationships between neurotransmitter function, alcohol, and sleep disturbances still require further elucidation.

—Kirk J. Brower

ReferencesAdamsonJBurdickJASleep of dry alcoholicsArchives of General Psychiatry281461491973468313910.1001/archpsyc.1973.01750310116019AldrichMSNeurobiology of sleepAldrichMSSleep MedicineNew YorkOxford19992738AsheychikRJacksonTBakerHThe efficacy of L-tryptophan in the reduction of sleep disturbance and depressive state in alcoholic patientsJournal of Studies on Alcohol505255321989268547110.15288/jsa.1989.50.525AubinHJGoldenbergFBenoitOEffects of tetrabamate and of diazepam on sleep polygraphy during subacute withdrawal in alcohol-dependent patientsHuman Psychopharmacology91911951994BeckerHCAlcohol withdrawal: Neuroadaptation and sensitizationCNS Spectrums4384057651999CarskadonMACavalloARosekindMRSleepiness and nap sleep following a morning dose of clonidineSleep123383441989276268810.1093/sleep/12.4.338EhlersCLAlcohol and sleepNoronhaAEckardtMWarrenKReview of NIAAA’s Neuroscience and Behavioral Research PortfolioNational Institute on Alcohol Abuse and Alcoholism Research Monograph No. 34NIH Publication No. 00–4520Bethesda, MDthe Institute2000417433FrenchTASegallMAWeinerNDevelopment of neurochemical and behavioral sensitivity to ethanol in long-sleep and short-sleep miceAlcohol124234311995851943710.1016/0741-8329(95)00025-mHawleyRJMajorLFSchulmanEALinnoilaMCerebrospinal fluid 3-methoxy-4-hydroxyphenylglycol and norepinephrine levels in alcohol withdrawal: Correlations with clinical signsArchives of General Psychiatry42105610621985405168310.1001/archpsyc.1985.01790340034005IshibashiMNakazawaYYokoyamaTCerebral atrophy and slow wave sleep of abstinent chronic alcoholicsDrug and Alcohol Dependence193253321987360879110.1016/0376-8716(87)90019-6JohnsonLCBurdickJASmithJSleep during alcohol intake and withdrawal in the chronic alcoholicArchives of General Psychiatry224064181970431430610.1001/archpsyc.1970.01740290022004JonesBEBasic mechanisms of sleep-wake statesKrygerMHRothTDementWCPrinciples and Practice of Sleep Medicine3d. edPhiladelphiaSaunders2000134154JouvetMBiogenic amines and the states of sleepScience16332411969430322510.1126/science.163.3862.32KoobGFRobertsAJBrain reward circuits in alcoholismCNS Spectrums423371999KruegerJMObalFJrFangJHumoral regulation of physiological sleep: Cytokines and GHRHJournal of Sleep Research8suppl 1535919991038910710.1046/j.1365-2869.1999.00009.xLandsWEAlcohol, slow wave sleep, and the somatotropic axisAlcohol1810912219991045656110.1016/s0741-8329(98)00073-1LejoyeuxMSolomonJAdesJBenzodiazepine treatment for alcohol-dependent patientsAlcohol and Alcoholism335635751998987234410.1093/alcalc/33.6.563LittletonJNeurochemical mechanisms underlying alcohol withdrawalAlcohol Health & Research World221324199815706728PMC6761820Mayo-SmithMFPharmacological management of alcohol withdrawal: A meta-analysis and evidence-based practice guideline. American Society of Addiction Medicine Working Group on Pharmacological Management of Alcohol WithdrawalJournal of the American Medical Association2781441511997921453110.1001/jama.278.2.144MunozCYojayRAcevedoXUse of antidepressant drugs in the study of the role of biogenic amines in ethanol narcosisActa Physiologica Et Pharmacologica Latinoamericana3631732719862953169Prospero-GarciaOCriadoJRHenriksenSJPharmacology of ethanol and glutamate antagonists on rodent sleep: A comparative studyPharmacology, Biochemistry and Behavior49413416199410.1016/0091-3057(94)90442-17824558RoehrsTRothTAlcohol-induced sleepiness and memory functionAlcohol Health & Research World191301351995PMC687572631798081RossettiZLLonguGMercuroGHmaidanYGessaGLBiphasic effect of ethanol on noradrenaline release in the frontal cortex of awake ratsAlcohol and Alcoholism2747748019921476551RouhaniSSantucciJDBajenaruOEffects of muscimol or homotaurine on sleep-wake states in alcohol-dependent rats during withdrawalPharmacology, Biochemistry and Behavior59955960199810.1016/s0091-3057(97)00521-29586855SharpleyALElliottJMAttenburrowMJCowenPJSlow wave sleep in humans: Role of 5-HT2A and 5-HT2C receptorsNeuropharmacology334674711994798428510.1016/0028-3908(94)90077-9SmithJWJohnsonLCBurdickJASleep, psychological and clinical changes during alcohol withdrawal in NAD-treated alcoholicsQuarterly Journal of Studies on Alcohol3298299419714332864ValenzuelaCFHarrisRAAlcohol: NeurobiologyLowinsonJHRuizPMillmanRBLangrodJGSubstance Abuse: A Comprehensive Textbook3d edBaltimoreWilliams and Wilkins1997119142WeinshenkerDRustNCMillerNSPalmiterRDEthanol-associated behaviors of mice lacking norepinephrineJournal of Neuroscience203157316420001077777910.1523/JNEUROSCI.20-09-03157.2000PMC6773122ZarconeVAlcoholism and sleepAdvances in Bioscience2129381978226436

The relationship between REM sleep indices and relapse has also been investigated in clinical outcome studies. In these studies, the sleep of recently abstinent patients is recorded during alcoholism treatment. Then, the patients are assessed several months later to determine if they resumed drinking during the followup period. Gillin and colleagues (1994) reported that increased REM%, shortened REM sleep latency, and possibly increased REM density were associated with higher relapse rates over a 3-month followup period in alcoholic patients without depression. Similarly, Brower and colleagues (1998) found that shortened REM sleep latency was associated with alcoholic relapse in nondepressed patients. Finally, in alcoholic patients with depression resulting from their alcohol use (i.e., secondary depression), increased REM density was associated with relapse at 3 months (Clark et al. 1998).

Thus, the results of clinical outcome studies suggest that increased pressure for REM sleep (i.e., *high* REM% and REM density as well as short REM sleep latency) is associated with relapse (Brower et al. 1998; Clark et al. 1998; Gillin et al. 1994). By contrast, the findings of experimental studies in animals and humans suggest that *low* REM% is associated with drinking and relapse (Aalto and Hilakivi 1986; Aalto and Kiianmaa 1986; Allen and Wagman 1975). Further work in both animals and humans is needed to reconcile the discrepancy in REM% findings.

Other clinical studies have implicated low amounts of SWS or SWS% as a marker of alcoholic relapse, although the evidence is relatively weak. Allen and colleagues (1977) performed sleep recordings on nine inpatients and later classified them as having either good or poor treatment outcomes based on amounts of sobriety over a 2-month followup period. The investigators found that patients with poor outcomes had significantly lower levels of baseline SWS% than did patients with good outcomes. Likewise, preliminary data collected by Aldrich and colleagues (1999) suggested that relapse was related to low levels of SWS%. However, these findings could not be replicated in a larger sample (Brower et al. 1998). Although the study by Brower and colleagues revealed low baseline levels in the proportion of stage 4 NREM sleep (rather than SWS sleep, which encompasses stages 3 and 4 NREM sleep) among patients who subsequently relapsed, the proportion of stage 4 sleep did not predict relapse after controlling for other sleep variables, such as sleep latency. Finally, several other clinical outcome studies detected no relationship between SWS measures and relapse (Clark et al. 1998; Drummond et al. 1988; Gillin et al. 1994).

Both objectively measured prolonged sleep latency and its subjective equivalent—self-reported difficulty falling asleep—also have been linked to relapse. With respect to objectively measured sleep, two polysomnographic studies reported a relationship between prolonged sleep latency at baseline and subsequent relapse (Brower et al. 1998; Drummond et al. 1998), whereas two other studies found no such relationship (Clark et al. 1998; Gillin et al. 1994). With respect to subjective measures, two recent studies of patients in alcoholism treatment found that subjectively measured difficulty falling asleep predicted relapse after 3 to 5 months (Brower et al. 1998; Foster and Peters 1999). Similarly, in the previously mentioned study by Skoloda and colleagues (1979) of alcoholic inpatients who could choose to drink after a 1-week baseline period of no drinking, patients who eventually drank reported more sleep problems at baseline, including difficulty falling asleep, than did nondrinking patients. Analyzed together, five of seven studies support a relationship between relapse and either prolonged sleep latency or difficulty falling asleep.

In addition to difficulty falling asleep, broader measures of subjectively reported insomnia also have been correlated with relapse (Brower et al. 2001; Foster and Peters 1999; Skoloda et al. 1979). Similarly, objective sleep measures, such as low levels of total sleep time (Clark et al. 1998) and sleep efficiency (Drummond et al. 1998) have been found to predict relapse. A history of using alcohol to promote sleep, however, does not appear to be a significant predictor of relapse (Brower et al. 2001).

#### Treatment Implications

If sleep problems are related to relapse, then treatment of sleep problems in alcoholic patients could possibly decrease relapse rates. Ordinarily, alcoholism treatment programs assume that sobriety is the best treatment for restoring a patient’s natural sleep rhythms. This treatment assumption is questionable, however, because as described earlier, multiple studies suggest that sleep disturbances can persist during abstinence and predispose patients to relapse. Some studies assessing the treatment of sleep disturbances in alcoholic patients have shown promise (Karam-Hage and Brower 2000; Longo and Johnson 1998; Greeff and Conradie 1998); however, these studies generally lacked control groups. In some cases, sedative drugs (i.e., benzodiazepines) have been used for treating sleep problems, but their use for this purpose remains controversial (Ciraulo and Nace 2000). To date, no controlled clinical trials have tested the hypothesis that treatment outcomes for alcoholism can be improved by concomitant treatment of sleep problems, and both pharmacological and nonpharmacological trials are warranted. The combination of several treatment approaches might be especially effective in this respect.

A brain chemical implicated in sleep regulation is melatonin. Supplemental melatonin has been used with mixed results to treat insomnia but appears most effective in people whose internal (i.e., endogenous) melatonin levels are low (Stone et al. 2000). Some studies suggest that melatonin levels are decreased in alcoholics (Schmitz et al. 1996; Wetterberg et al. 1992); accordingly, supplemental melatonin could be investigated for the treatment of alcoholic patients with both insomnia and low levels of endogenous melatonin.

## Summary

Sleep problems are common, potentially fatal, and costly among alcoholics. Sleep problems may occur during active drinking, acute alcohol withdrawal, and protracted withdrawal. Although most sleep abnormalities improve over time, some problems persist for months to years after initiating abstinence. Disturbances of sleep may either precede or follow the development of alcoholism. Whether sleep disturbances predispose some children and adults to develop abnormal patterns of drinking is unknown. Some evidence suggests that alcohol is more reinforcing in non-alcoholic people with insomnia than in people without insomnia, suggesting an increased likelihood of alcohol use in people with insomnia. Similarly, people with insomnia are more likely to report using alcohol to aid sleep than are people without insomnia. The use of alcohol to self-medicate sleep problems is especially common, although not particularly effective, among alcoholics.

Several studies during the past 25 years have demonstrated a relationship between baseline sleep problems when patients enter alcoholism treatment and subsequent relapse to drinking. Sleep predictors of relapse include insomnia, especially difficulty falling asleep, and various polysomnographic abnormalities, such as increased sleep latency, REM%, and REM density as well as decreased SWS, REM sleep latency, sleep efficiency, and total sleep time. Uncontrolled observations of treating alcohol-related insomnia with either medication or behavioral therapy appear promising, but the hypothesis that effective treatment of sleep problems reduces relapse rates in alcoholics warrants testing in controlled clinical trials.

## Figures and Tables

**Table 1:** Summary of Studies Determining Rates of Insomnia in Alcoholics

Author(s) (year)	Sample	Sleep Measure	Rate of Insomnia (%)	Relation to Last Drink
Mello and Mendelson (1970)	40 male inpatients	Behavioral observation	58*	During the first 6 days of acute alcohol withdrawal
Baekeland et al. (1974)	294 outpatients	4-point, single-item rating scale	36	At time of admission after 9.3 (± 22.8) days of abstinence; 43 percent had 0 days of abstinence
Feuerlein (1974)	112 inpatients and 72 outpatients	Semi-standardized interview	37	Not specified
Caetano et al. (1998)	748 men in public detoxification or residential treatment	Standardized questionnaire	67	During acute alcohol withdrawal
Foster et al. (2000)	41 male and 41 female inpatients	7-point, single-item rating scale	72	At 4 to 5 days after detoxification
Brower et al. (2001)	146 male and 26 female inpatients and outpatients	8 items from the Sleep Disorders Questionnaire	61	During 6 months prior to admission

* After excluding five patients whose insomnia may have been caused by the withdrawal of medication for detoxification, the rate of insomnia was 45 percent (see text of main article).

**Table 2:** Sleep Disturbances in Alcoholic Men During Sustained Abstinence as Determined in Polysomnographic Studies

Study	Sample (design)	Abstinence Duration	Results
Adamson and Burdick (1973)	10 subjects recruited from AA and 10 normal control subjects (cross-sectional)	1–2 yr	↑ stage changes, but no subjective complaints and no abnormalities of SL, TST, SE, REM%; ↓ stage 4% (*p* < 0.10)
Wagman and Allen (1975)	20 subjects recruited from AA (cross-sectional)	200 wk (~ 4 yr)	SWS% normal
Williams and Rundell (1981)	46 alcoholics and 20 control subjects (longitudinal)	3 mo	↑ SL, stage 1%, REM%, stage changes, arousals, REM disruptions; ↓ stage 2%, SWS%, REM latency
	24 alcoholics	9 mo	SL, REM% normal; ↑ stage 1%, stage changes, arousals, REM disruptions; ↓ stage 2%, SWS%, REM latency
	5 alcoholics	21 mo	SL, stage 1%, REM%, SWS% normal; ↑ stage changes, arousals, REM disruptions
Imatoh et al. (1986)	6 alcoholics (longitudinal)	3 mo	REM% normal; ↓ SWS%, REM latency
Ishibashi et al. (1987)	5 alcoholics (longitudinal)	6 mo	↓ SWS% associated with cerebral atrophy
Drummond et al. (1998)	29 alcoholics and 28 control subjects (longitudinal)	5 mo	SL and SE normal; ↑ REM%; ↓ TST, SWS%, REM latency
	9 alcoholics	14 mo	SL, SE, and TST normal; ↑ REM%; ↓ SWS%, REM latency
	4 alcoholics	27 mo	SL, SE, TST, and SWS% normal; ↑ REM%; ↓ REM latency
Schiavi et al. (1995)	20 alcoholics and 20 control subjects	2–36 mo	SL, REM% normal

AA = Alcoholics Anonymous; mo= months; REM% = percentage of rapid eye movement sleep; SE = sleep efficiency; SL = sleep latency; SWS% = percentage of slow-wave sleep; TST = total sleep time; wk=weeks; yr=years; ↑ = increase; ↓ = decrease. NOTES: Cross-sectional studies evaluate each participant at only one point in time; longitudinal studies follow the same participants over an extended period of time.

## Footnotes

1 For a definition of this and other terms used in this article, see the glossary, p. 125.

2 For these experiments, alcoholics undergoing inpatient treatment typically were given alcohol for 1 or more days before withdrawal was induced by withholding alcohol. Such an experimental design raises ethical issues by current standards of human research and is generally no longer being used.

3 In that study, participants drank 8.6 ounces of pure alcohol for 5 days followed by 11.4 ounces of pure alcohol for 5 days. One standard drink (i.e., 12 ounces of beer, 5 ounces of wine, or 1.5 ounces of 80 proof distilled spirits) contains approximately 0.5 ounce of pure alcohol.

## GLOSSARY

Brain stem: The oldest brain area, located at the base of the brain; performs motor, sensory, and reflex functions.
Cerebral cortex: The intricately folded outer layer of the *cerebrum*. Composed of nerve cells (i.e., neurons), the cortex contains areas for processing sensory information and for controlling motor functions, speech, higher cognitive functions, emotions, behavior, and memory.
Cerebrospinal fluid: Fluid that surrounds the brain and the spinal cord as well as fills the chambers within the brain.
Cerebrum: The largest portion of the brain; includes the cerebral hemispheres.
Insomnia: (1) A patient’s subjective complaint of having difficulty falling and/or staying asleep or of experiencing non-restorative sleep (i.e., feeling not rested in the morning). (2) A *sleep disorder* characterized by insufficient or nonrestorative sleep for multiple, consecutive nights that results in significant daytime distress or impaired functioning.
Neurotransmitter: A chemical messenger released by an excited or stimulated nerve cell and used to transmit nerve signals from one nerve cell to the next. These messengers can be categorized into one of three classifications: (1) stimulatory neurotransmitters, which cause the generation of a new nerve signal; (2) inhibitory neurotransmitters, which prevent the formation of a new nerve signal; and (3) modulatory neurotransmitters, which affect biological activity in the cell.
Nonrapid eye movement (NREM) sleep: Consists of four stages. Stages 1 and 2 sleep are sometimes referred to as light sleep, because people are relatively easy to awaken during these stages. Conversely, stages 3 and 4 sleep are referred to as deep sleep or *slow-wave sleep (SWS)*, because people are most difficult to arouse during these latter stages.
Periodic limb movement (PLM) disorder: A disorder characterized by repetitive jerking of the legs and sometimes the arms during sleep. Multiple arousals from sleep caused by these sudden movements result in *insomnia* or feelings of sleepiness during the daytime.
Polysomnography: The gold standard for objectively measuring sleep characteristics during the night. Includes measures of breathing disturbances, abnormal leg movements, eye movements, and brain-wave activity. Specific measures include the percentage of time spent in each sleep stage (i.e., *REM sleep* and stages 1, 2, 3, and 4 of *NREM sleep*); sleep continuity (i.e., sleep latency, total sleep time, and sleep efficiency); and *REM sleep latency*.
Rapid eye movement (REM) sleep: A light sleep phase characterized by rapid movements of the eyes; is commonly associated with dreaming. Acute alcohol intoxication is typically associated with REM sleep suppression, whereas alcohol withdrawal is associated with REM rebound.
Receptor: A protein molecule usually found on the surface of a neuron or other cell that recognizes and binds to *neurotransmitters* or other chemical messengers.
REM%: The percentage of total sleep time spent in REM sleep.
REM sleep latency: The time between the onset of sleep and the onset of the first REM episode. Acute alcohol intoxication is typically associated with prolonged REM sleep latency, whereas alcohol withdrawal is associated with short REM latency.
Sleep apnea: A *sleep disorder* characterized by recurrent episodes of breathing cessation during the night associated with short arousals—which the patient usually does not remember—that enable the patient to resume breathing. These multiple arousals can result in *insomnia* or feelings of sleepiness during the daytime.
Sleep disorder: A constellation of sleep problems and associated features that is detrimental to a person’s health, such as *sleep apnea* and *periodic limb movement disorder*. Sleep disorders are diagnosed by specific criteria and are often treatable.
Sleep problems: A general term that describes any subjectively perceived or objectively measured problem with sleep (e.g., complaints of *insomnia* or an increase in *REM%*). Also referred to as sleep disturbances, abnormalities, impairment, and disruption. Sleep problems are more common in alcoholics than in nonalcoholics.
Slow-wave sleep (SWS): A collective term for stages 3 and 4 of *NREM sleep*. Acute alcohol intoxication is typically associated with an increase in SWS, whereas SWS in alcoholics is markedly reduced during abstinence.
SWS%: The percentage of total sleep time spent in *SWS*.
